# Optogenetic Investigation of Arousal Circuits

**DOI:** 10.3390/ijms18081773

**Published:** 2017-08-15

**Authors:** Susan M. Tyree, Luis de Lecea

**Affiliations:** Department of Psychiatry and Behavioral Sciences, Stanford University, 1201 Welch Road, Stanford, CA 94305, USA; suszie.tyree@gmail.com

**Keywords:** optogenetics, arousal, sleep-wake transitions, hypocretin, circuit investigation

## Abstract

Modulation between sleep and wake states is controlled by a number of heterogeneous neuron populations. Due to the topological proximity and genetic co-localization of the neurons underlying sleep-wake state modulation optogenetic methods offer a significant improvement in the ability to benefit from both the precision of genetic targeting and millisecond temporal control. Beginning with an overview of the neuron populations mediating arousal, this review outlines the progress that has been made in the investigation of arousal circuits since the incorporation of optogenetic techniques and the first in vivo application of optogenetic stimulation in hypocretin neurons in the lateral hypothalamus. This overview is followed by a discussion of the future progress that can be made by incorporating more recent technological developments into the research of neural circuits.

## 1. Introduction

Before the development of optogenetic tools researchers used methods such as electrophysiological stimulation and recording of neural activity, pharmacological stimulation or inhibition of targeted neuron populations, or the manipulation of gene expression through use of knockout animals to investigate the function and activation patterns of different neural populations. While these methods have undoubtedly produced an immense body of literature and insight into neural functions, structures, and mechanisms across multiple areas of neuroscience research, as new technologies have developed, the weaknesses of these techniques have naturally become highlighted. While electrophysiology allows the temporal precision that is lacking in pharmacological and genetic manipulation, it lacks the ability to target specific neuron populations using their gene expression. Pharmacological and genetic manipulations allow this neuron targeting specificity but lack the temporal flexibility of electrophysiological recordings, either requiring time for the pharmacological agent to peak or washout, or, in genetically manipulated mice, lacking the ability to observe the effect of the gene knockout during specific development periods, or even during specific behaviors.

The development of optogenetic tools has provided researchers with a method to genetically target specific neuron populations with millisecond precision, either for recording (fiber-photometry) or stimulation (optogenetic activation) of neural activity. The advances provided by this technique have allowed researchers to combine the gene-targeting specificity of pharmacological and genetic manipulations with the temporal flexibility of electrophysiological recordings. While the use of electrophysiological recording and stimulation methods is still very useful for applications where the genetic profile of the target neurons are not yet known or are not uniform, research areas in which it is known which neurotransmitters and cell types are involved can benefit greatly from this specificity. One area of research that has benefited greatly from this precision and flexibility is the investigation of neural circuits underlying arousal and transitions between sleeping and waking states. This review will focus on the neural correlates of sleep-wake state modulation, giving an overview of the neural populations involved, and presenting the progress that has been made following the incorporation of optogenetic technologies into the investigation of individual neuron populations, as well as circuit integration.

## 2. The Importance of Sleep

Considering the ubiquity of sleep across the animal kingdom, the precise function and purpose of sleep remains relatively elusive; however, it is apparent that effectively regulating arousal is crucial for survival. As well as maintaining an appropriate sleep/wake cycle we must also be able to respond appropriately to unexpected stimuli, whether they be an unanticipated reward or the sudden presentation of a stressor. The ability to focus and react can mean the difference between life and death. In states of disordered arousal we may exhibit hyper-arousal—the inability to drop out of a state of heightened vigilance resulting in anxiety disorders, insomnia, etc. or if we are unable to successfully maintain stable sleep-wake states this can lead to either an inability to maintain prolonged sleep or prolonged wakefulness. In order to understand what is going wrong in sleep disorders, researchers first had to understand what normal, healthy sleep-wake states look like.

### Defining Different Sleep-Wake States

The first step away from a binary “sleep” versus “wake” definition of sleep-wake states came with the observation of what is now called Rapid Eye Movement (REM) sleep, in which a sleeping subject elicits eye movements and electroencephalogram (EEG) activity that is similar to those elicited from an awake subject. REM sleep was first observed by Aserinsky and Kleitman [1], and this observation lead to the categorization of sleep into two states: REM and non-REM (NREM). In order to study the neurocircuitry underlying different these sleep-wake states it is first important to be able to characterize them. For this purpose sleep-wake states have been broadly categorized into four different states: active/motivated wakefulness, quiet wakefulness, NREM sleep, and REM sleep. In the laboratory, researchers use EEG and electromyogram (EMG) recordings to distinguish between these different sleep-wake states [2]. Broadly, sleep/wake states are categorized as:

- Active/motivated wakefulness—θ (5–9 Hz) [3,4] and γ (40–200 Hz) [5] EEG oscillations paired with muscle activity shown in the EMG signal;
- Quiet wakefulness—slower EEG frequencies such as α (8–14 Hz) and β (15–24 Hz) [6] and muscle activity observed in the EMG signal;
- NREM—high amplitude, low frequency Δ oscillations (0.5–4 Hz) [7,8,9,10,11] and spindles (bursts of 7–15 Hz oscillations) [12,13,14], and decreased muscle activity;
- REM—dominated by θ oscillations and strong suppression of muscular tone [15,16,17,18].

Alongside these sleep-wake states, further categorizations are possible to perform more fine-grained analysis of different sleep states, for example in humans NREM sleep can be further categorized into four different stages (I–VI) of increasing depth of sleep [2]. However for the sake of this review a broader categorization of REM, NREM, waking, and occasional specifications of active waking and hyper-arousal states will be used to discuss the neural mechanisms linked to these states.

## 3. Neural Correlates of Sleep-Wake Modulation

Researchers studying sleep to wake transitions have used many different methods to uncover the numerous neuron populations that are involved in modulating wakefulness and sleep states. Following the influenza epidemic of 1918, Economo [19] performed post-mortem examinations of the brain tissue of patients who had suffered excessive sleepiness after contracting the influenza virus. It was observed that this sleepiness was correlated to brain lesions in the posterior hypothalamus. As well as specific brain areas, human responses to medications and disease states have also implicated specific neuropeptides in the modulation of sleep and wake states. Post-mortem examinations have shown a substantial loss of hypothalamic hypocretin (also known as orexin) neurons in the brains of patients with narcolepsy [20,21], a sleep disorder characterized by excessive sleepiness. Whereas a role for dopamine in modulation of arousal states has been implicated following an observation that patients with Parkinson’s disease, which results in the degeneration of dopaminergic neurons in the substantia nigra (among other neurological changes), experience sleep disturbances such as excessive sleepiness during the day and difficulty sleeping through the night [22].

**Figure 1:**
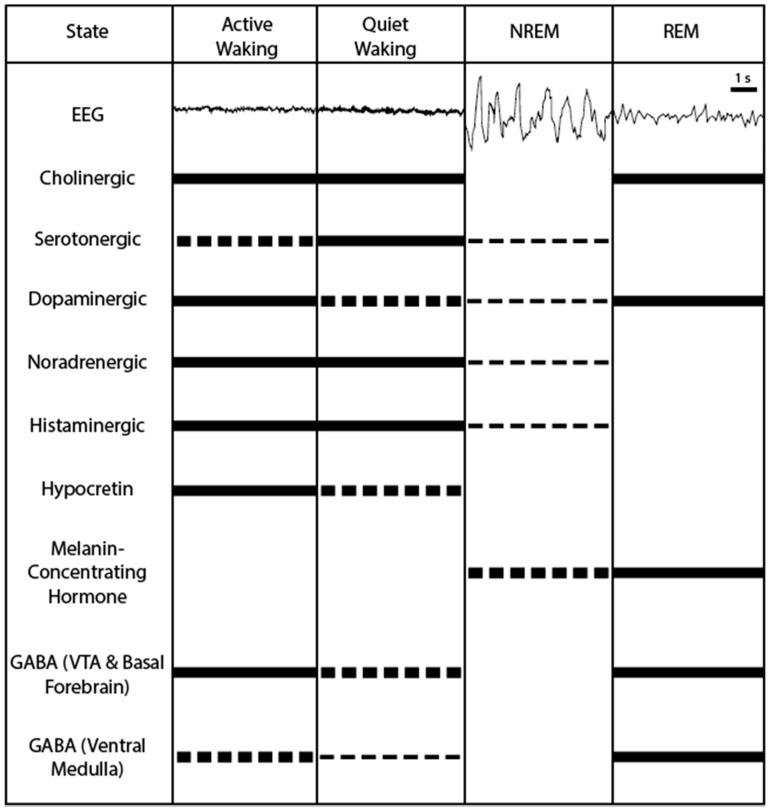
A visual representation of neural activity profiles of sleep/wake-active neurons populations. Showing high (solid line), moderate (thick broken line), low (thin broken line), and quiet (no line) activity profiles during active waking, quiet waking, non-rapid eye movement sleep (NREM), and rapid eye movement sleep (REM) states in basal forebrain [25,26,27,28,29,30] and brainstem [31,32,33,34,35] cholinergic, dorsal raphe nuclei serotonergic [36,37,38], ventral tegmental area (VTA) dopaminergic [39,40], locus coeruleus noradrenergic [41,42,43,44], tuberomammillary nucleus histaminergic [45,46,47], lateral hypothalamic hypocretin [48,49,50,51,52,53,54,55,56], lateral hypothalamic area melanin-concentrating hormone [57,58,59,60], VTA and basal forebrain γ amino butyric acidergic (GABAergic) [26,61], and ventral medulla GABAergic neurons [62]. 1 s = 1 second.

Pharmaceuticals have also highlighted potential candidate systems; particularly the development of first-generation antihistamines (histamine H_1_ receptor antagonists) and their sedative side effects implicated histamine as a wake-promoting neuropeptide [23,24]. In order to further investigate these neuropeptides and neural structures under more strictly controlled conditions researchers shifted into animals models to observe the mechanisms underlying sleep-wake state modulation on a more fine-grained, cellular level. Although there are some notable differences between humans and other animals when it comes to sleeping patterns, animal models of sleep have proved to be useful for further unraveling the neural circuits involved in modulating sleep and waking states. The use of animal models, particularly rodent models have resulted in the identification of multiple neuropeptides and structures that can be targeted to modulate sleep to wake states. These findings are reviewed below, with an overview of activity patterns of these candidate neurons across sleep-wake states shown in Figure 1.

### 3.1. Cholinergic Neurons: Active during Waking and REM Sleep

Cholinergic neurons have been shown to modulate arousal, in particular the basal forebrain and the mesopontine tegmentum of the brainstem contain neurons that release acetylcholine and modulate sleep-wake states [63,64]. Cholinergic neurons in the basal forebrain are most active during wakefulness and REM sleep [25,26,30], with a high correlation between their firing rate and cortical activation [25,65,66]. This pattern of wake-active and REM-active cholinergic neurons has also been observed in populations of cholinergic neurons found in the mesopontine tegmentum [31,32,33,34,35] and is supported by evidence of higher levels of acetylcholine in cortical and thalamic areas during wakefulness and REM sleep [27,28,29]. Recordings of cholinergic neurons have also shown that they fire in response to cortical activity occurring following tail-pinch in urethane-anaesthetized animals [67]. While these results suggest a role for cholinergic neurons in processes underlying wakefulness and REM sleep, lesion studies have returned mixed results. Some studies using selective lesions of cholinergic neurons in the basal forebrain only produced minor changes to the wakefulness of the animals [68,69,70], which would suggest that these neurons are not necessary to maintain wakefulness. However, more extensive lesions of cholinergic neurons in the basal forebrain resulted in a reduction of high-frequency EEG power, with a particularly noticeable reduction in γ-activity [71,72], which occurs during active-waking. These more extensive lesions also reduced the normal homeostatic responses to increased sleep-drive following sleep deprivation [73,74,75]. A pharmacological study which infused cholinomimetics into the pontine reticular formation, showed that increasing cholinergic activity in the reticular formation resulted in increased REM sleep [76], which would suggest a REM-sleep promoting role for brainstem cholinergic neurons. The heterogeneity of the basal forebrain and brainstem areas where these cholinergic neurons are found make these populations ideal candidates for genetically targeted optogenetic investigation to further explore these divergent functions of sleep-promoting basal forebrain cholinergic neurons, and REM-promoting brainstem cholinergic neurons.

### 3.2. Serotonergic Neurons: Promoting Wakefulness

Serotonergic interactions with sleep came into the spotlight due to the changes of sleep architecture seen in depressed patients undergoing pharmaceutical treatments affecting the serotonin system, such as a significant decrease in REM sleep duration in patients undergoing treatment with tricyclic antidepressant amitriptyline [77]. In line with this finding, systemic administration of serotonin receptor agonists produces a reduction in NREM and REM sleep and promote waking [78]. Additionally, while normal mice usually exhibit a boost in REM following immobilization stress, this REM rebound is not observed in 5-HT_1A_ knockout mice [79]. Electrophysiological recordings have shown that serotonin is most active during waking, less active during NREM, and quiet during REM [80,81], these findings have also been supported by studies measuring the release of serotonin [37,38]. Serotonin neurons are activated by stressful stimuli [82], however they are most active during feeding and quiet waking, and are less active during active-waking [36], suggesting a possible role for serotonin in promoting a state of quiet-waking.

### 3.3. Noradrenergic Neurons: Promoting Wakefulness

The LC is the origin of the majority of noradrenergic projections of the forebrain, others arise from small clusters of norepinephrine (NE) neurons throughout the brainstem [83]. Administration of norepinephrine into the forebrain ventricles results in increased wakefulness [84,85]. LC neurons are most active during waking, particularly when the animal is exposed to stressful stimuli [41], they are less active during NREM sleep, and quiet during REM sleep [42,43,44]. Despite this clear link between LC/NE activity and wake states, lesion studies have produced minimal effects on total waking time in LC lesioned animals [86] and increases in REM sleep [87] and reduced expression of wake-related gene transcripts [88,89,90] following norepinephrine depletion. Discrepancies between the pharmacological studies, electrophysiological recordings, and lesion studies could suggest that while norepinephrine activation in the LC is sufficient to induce wakefulness, these neurons are not necessary to maintain wake-states.

### 3.4. Dopaminergic Neurons: Promoting Wakefulness

The role of dopamine in modulating wakefulness came into the spotlight due to the side effect of increased alertness following the consumption of certain drugs of abuse that are categorized as stimulants. Stimulants such as amphetamine and modafinil interact with other neuromodulatory systems, as well as dopaminergic neurons they also enhance the release of other wake-promoting neuropeptides serotonin and norepinephrine. In order to determine the main mechanism inducing increased alertness Wisor, et al. [91] used a dopamine transporter knockout mouse to show that animals lacking dopamine transporter do not exhibit the stimulant effects of modafinil or amphetamine, suggesting that transportation of dopamine is necessary for these stimulant effects. Interestingly, knockout studies have produced mixed results, with knockouts of the D2 receptor exhibiting bouts of waking, overall decreased waking amounts, and increased sleep duration [92], whereas dopamine transporter knockout mice exhibit reduced NREM sleep and increased wakefulness consolidation [91]. This suggests that dopamine may exhibit more nuanced modulation of sleep-wake states.

Dopaminergic (DA) neurons in both the ventral tegmental area (VTA) and the ventral periaqueductal gray matter (vPAG) have been investigated to observe their activity patterns across different sleep/wake states. Fos studies have shown that DA neurons in the vPAG are active during waking but not sleep states [93], however DA neurons in the VTA and the substantia nigra have a relatively consistent firing rate across all sleep-wake states [94]. An observation of burst-activity revealed that, despite the consistency of firing rates in the VTA, there were a higher number of DA neurons firing bursts in waking and REM sleep as opposed to NREM sleep, which caused increased levels of DA in the prefrontal cortex, nucleus accumbens, and other targets of VTA^DA^ neurons [39]. Additionally, Steinfels, et al. [95] showed that increased in DA bursting correlated strongly with salient stimuli that were either aversive or rewarding, suggesting that VTA^DA^ neurons may play a role in the directing of attention. The case for DA modulation of arousal is also supported by lesion studies in the vPAG, which showed a ~20% reduction in waking duration [93]. The variations in activity profiles suggest that these wake-active vPAG^DA^ neurons are performing separate functions to the consistently active VTA^DA^ neurons that elicit increased burst activity during waking and REM states.

### 3.5. Neuropeptide S: Promoting Wakefulness

Neuropeptide S (NPS) precursor mRNA (messenger ribonucleic acid) is most strongly expressed around the locus coeruleus (LC), in the the principal sensory 5 nucleus, and in the the lateral parabrachial nucleus, and its receptor mRNA has highest expression levels in the cortex, thalamus, hypothalamus, and amygdala [96]. NPS has been proposed as a mediator of anxiety-related behaviors following evidence that intracerebroventricular (i.c.v.) administration of NPS reduces behavioral markers of anxiety in standard tests including the elevated plus maze and open field [96]. Additionally, considering that i.c.v. NPS administration also produces increased locomotion and reduced the amount of time animals spent in paradoxical REM sleep and the deepest stage of NREM sleep known as slow wave sleep [96]. Alternatively, knocking out the NPS receptor resulted in a decrease explorative behaviors in a novel environment [97], suggesting increased anxiety. Due to these findings it has been suggested that NPS may play a role in modulating states of hyper-vigilance such as fear or anxiety [96,98]. Due to the proximity of NPS to other neurons known to modulate sleep-wake states, such as noradrenergic neurons in the locus coeruleus and neuron populations in the parabrachial nucleus, these pharmacological methods have been used to avoid conflating the effects of unintentional activation of these neighboring populations to NPS.3.6. Histaminergic neurons: promoting wakefulness.

The link between histamine and wake-promotion was first suggested due to the side effect of drowsiness caused by first-generation antihistamines, which antagonized histamine H_1_ receptors [23,24]. A wake-promoting population of histaminergic neurons is located in the tuberomammillary nucleus, these neurons are most active during waking, less active during NREM sleep, and quiet during REM sleep [45,46,47]. Despite the obvious sedative effect of H_1_ receptor antagonists, as well as subsequent studies showing that pharmacological inhibition of the histamine system produces drowsiness whereas pharmacological activation of the histamine system promotes wakefulness [23,99], other investigations of histaminergic modulation of wakefulness have produces mixed results. Lesion studies in the cat [100] and the rat [101] produced no effect on sleep/wake duration, suggesting that this system is not critical for the maintenance of sleep/wake architecture. H_1_ receptor knockout mice exhibited similar sleep/wake durations to controls, only showing fewer micro-arousals (<16 s brief awakening), and fewer transitions between NREM sleep and REM sleep, but no difference in overall sleep/wake duration [102]. Investigation of the histamine system using a knockout model where the histamine-synthesizing enzyme histidine decarboxylase is knocked out (HDC KO) did not change the duration of sleep/wake states under baseline conditions, however HDC KO animals did show decreased wakefulness in a novel environment paradigm [103,104], suggesting a possible role for histamine in hyper-vigilance required in the presence of novel stimuli [23].

### 3.6. Hypocretin Neurons: Promoting Wakefulness

Hypocretin (Hcrt) neurons are found in the lateral hypothalamus and were first linked to sleep function due to a significant loss of Hcrt neurons in the brains of patients with narcolepsy [20,21] and low levels of Hcrt-1 in the cerebrospinal fluid (CSF) of narcoleptic patients [105]. The role of Hcrt in narcolepsy was further supported by animal models such as canine narcolepsy, which was linked to a mutation in the Hcrt 2 receptor [106]. Similarly, mouse models, which present both behavioral and EEG signs of narcolepsy, have been developed by manipulating the Hcrt system: either by targeting the neuropeptide in a Hcrt knockout mouse line [107] or by genetically ablating the neurons themselves [108].

Studies investigating the role of Hcrt in sleep modulation in non-diseased mouse models have found evidence for a wake-promoting role for Hcrt; Hcrt 1 administration (i.c.v.) increases wakefulness in a dose-dependent manner in rats [109]. Conversely, pharmacological antagonism of the Hcrt receptors results in increased NREM and REM sleep and reduced wakefulness in both animals and humans [110], and inhibition of Hcrt neurons using Designer Receptors Exclusively Activated by Designer Drugs (DREADDs) promotes sleep [111]. Hcrt neurons are most active during active-waking, less active during quiet-waking, and are quiet during sleep, this has been shown in Fos studies [48,49], measurements of Hcrt peptide release [50], and single-unit recordings [51,52,53,54,55]. Additionally, Hcrt levels have been shown to peak toward the end of the day in squirrel monkeys, whose sleep-cycle is similar to that of humans, suggesting that as the wake-cycle progresses wake-promoting Hcrt activity increases, perhaps in opposition to an increased need to rest [56]. Taken together along with the evidence from narcoleptic patients showing reduced ability to maintain wakefulness, these findings suggest a role for Hcrt in maintaining wakefulness, particularly toward the end of the waking-period of the animal.

While considering the role of different neuropeptides in modulating sleep-wake states across the day/night cycle, researchers must also consider how outside influences on sleep behaviors interact with these neurotransmitters. The two strongest determinants of sleeping behaviors are time of day and food availability, humans generally adhere to an active light-period and a quiet dark-period. EEG rhythms that are used to distinguish between sleep-wake states can oscillate according to these 24 h light–dark cycles, for example hippocampal θ rhythm, most often associated with waking states, is modulated on a circadian rhythm and can be entrained by food availability [112]. Considering evidence that genetic ablation of Hcrt neurons results in reduced mRNA expression of numerous clock genes in the forebrain and restricted feeding can shift the peak of Hcrt activity [113], it is possible that Hcrt may play a role in mediating food-related shifts in circadian rhythms, and thus effect sleeping behaviors. This idea is supported by evidence suggesting that nutritional status can affect Hcrt activity, in particular, wake-active Hcrt neurons are inhibited following food-intake and Hcrt neuron activity increases during periods of fasting in non-human primates [114]. Additionally, normal behavioral responses to fasting, such as increased waking and foraging behaviors, are not observed in mice with a genetic ablation of Hcrt neurons [113,115,116], suggesting that Hcrt is not only involved in, but also necessary for these behavioral responses to food availability. Mechanistically there are many candidates to investigate how Hcrt activity is modulated by food intake, Hcrt neurons interact with multiple biomarkers related to nutritional state, and in particular appear to be inhibited by biomarkers whose release is triggered by food intake, such as leptin, glucose, and neuropeptide Y [115,116,117,118,119,120]. Taken together these findings suggest a role for Hcrt in modulating not only sleep-wake transitions, but also more general sleeping behaviors such shifting circadian rhythms in response to changes in food availability or nutritional status.

### 3.7. Melanin-Concentrating Hormone Neurons: Promoting REM Sleep

Melanin-concentrating hormone (MCH) is another sleep-relevant neuron population located in the hypothalamus. MCH appears to have an opposing function to the other hypothalamic population of Hcrt neurons, while Hcrt promotes wakefulness it appears that MCH plays a role in promoting sleep, particularly REM sleep. Fos studies have shown that MCH neurons are most responsive after REM sleep [57,58]. This finding has been supported by a study using cellular recordings, which observed that MCH neurons only fire during REM sleep [59]. Observation of MCH levels across the sleep-wake cycle in the amygdala of humans also showed that the onset of sleep produces an increase in MCH levels [60]. In order to further explore the role of MCH in sleep-wake states researchers have also performed a variety of manipulation studies such as administration of MCH (i.c.v.) at the beginning of the light period (sleep phase for rodents), which showed a dramatic ~300% increase in the amount of REM sleep, and a slightly lower~150% increase in NREM sleep, as opposed to controls [58]. Concordantly, administration of an MCH antagonist decreases the amount of REM and NREM sleep [121], together these findings support the idea of MCH being a sleep-promoting neuropeptide. These findings would suggest that any sleep-relevant function of MCH neurons must be involved in REM sleep, however studies using genetic manipulation to investigate MCH function have returned mixed results. MCH receptor 1 knockout animals showed no change in sleep levels and no change in sleep rebound following a sleep deprivation paradigm [122], whereas MCH knockout mice showed minor decreases in sleep duration at baseline conditions, but showed a significant decrease in REM sleep and an increase in behavioral hyperactivity in response to fasting [123]. This link to fasting has also been supported by in vitro evidence that MCH neurons can be excited by increased circulating glucose levels [124]. This evidence for a modulatory effect of MCH function could suggest a possible mechanism for the modulatory effect of nutritional status on sleep function and appears to be in opposition to the effects seen in Hcrt neurons.

### 3.8. Glutamatergic and GABAergic Neurons

Alongside the other neuropeptides mentioned researchers must also consider the contribution of glutamatergic neurons and γ-amino butyric acidergic (GABAergic) neurons to sleep-wake states. These neuron types are found in all of the brain areas previously discussed [125,126,127,128,129,130,131], and in some cases are co-expressed in the same neurons with these other neuropeptides. For example, Vincent, Hokfelt, Skirboll and Wu [130] observed neurons co-expressing GABA and histamine in the hypothalamus, this has also been observed with GABA and MCH [129], and it has been suggested that glutamate may be a co-transmitter of Hcrt neurons in the hypothalamus [132], NPS neurons near the LC [96,133], and serotonin neurons in the dorsal raphe nuclei [134]. Additionally, animals with Vglut2 (vesicular glutamate transporter 2) deletion in the lateral parabrachial nucleus exhibited impaired ability to arouse from sleep and animals with the same treatment in the medial parabrachial nucleus exhibited shorter waking periods (~20%) and longer NREM duration (~43%) [135], suggesting a role for glutamatergic parabrachial nucleus neurons in modulating sleep–wake states.

Within the hypothalamus it has been shown that a population of wake-active neurons will increase their firing rate in response to antagonism of GABA_A_ receptors [136], whereas GABAergic neurons in the BF and VTA show increased firing during waking and REM sleep [26,137]. It is thought that these VTA^GABA^ neurons in particular may be involved in directing arousal linked to the reward system following evidence that their firing increases before intracranial self-stimulation in the medial forebrain bundle [61]. GABAergic neurons in the preoptic area have also been put forward as a candidate for mediating sleep-wake states. Electrophysiological studies have shown that these preoptic area neurons are predominantly sleep-active, and lesions of the ventrolateral preoptic area results in insomnia [138]. Additionally, GABAergic neurons have also been shown to project to the lateral hypothalamic area from the ventrolateral preoptic area and median preoptic nucleus [139]. These opposing actions of the hypothalamus and the preoptic area, coupled with the synaptic connections between the two structures have lead researchers to propose a possible state-switch mechanism, whereby the sleep-promoting preoptic area and the wake-promoting hypothalamus communicate to effectively switch between sleep-wake states [43]. The heterogeneity of these neuron populations makes functional investigation using traditional methods such as lesions problematic, as it is difficult to know if the effects are due to lesioning/recording/stimulating the other sleep–wake-relevant neuron populations that are known to be neighboring, or even co-expressed within, the neurons being targeted.

## 4. Optogenetic Investigation of Individual Neuron Populations

The development of optogenetics has revolutionized neuroscience research. It has been particularly useful for studying heterogeneous neuron populations in areas like the hypothalamus where many neuropeptides may be co-expressed within the same cell. Within the hypothalamus investigation of Hcrt and MCH neurons were made particularly difficult using traditional research methods due to their proximity and their apparent duality of function [59]. The gene-targeting specificity permitted by optogenetics has allowed researchers to overcome these problems studying sleep circuits.

### 4.1. Hypocretin Neurons

The first illustration of in vivo optogenetic manipulation was an investigation of Hcrt activation in the lateral hypothalamus (LH), which showed that optogenetic activation (5–30 Hz) of Hcrt neurons increases the probability of a sleep-wake transition from either NREM or REM sleep [140]. This effect was apparent throughout both light and dark phases unless the animal was placed in a 2 or 4 h sleep-deprivation paradigm resulting in increased sleep pressure [141]. Taken together these results suggest that Hcrt activity may modulate sleep-wake transitions according to homeostatic sleep-need. While these optogenetic stimulation studies have investigated the effect of activating Hcrt neurons [142,143] have also investigated the effect of optogenetically silencing Hcrt neurons using two transgenic mouse lines. Optogenetic inhibition of Hcrt neurons during the sleep-phase in mice expressing halorhodopsin in Hcrt neurons resulted in EEG and EMG patterns characteristically seen during NREM sleep, whereas no effect was seen following inhibition during the wake-phase [142]. Using a second transgenic mouse line with archaerhodopsin expressed in Hcrt neurons, [143] found that 1 h of Hcrt inhibition during the wake-phase can increase the total amount of time spent sleeping and reduce the total waking-time. These experiments have shown that stimulating Hcrt neurons can increase the likelihood of transitions into wakefulness, whereas inhibition of these neurons increases the likelihood of transitions into sleeping states.

### 4.2. Melanin-Concentrating Hormone Neurons

Optogenetic techniques have also been used to investigate MCH neurons in the hypothalamus showing that optogenetic activation of LH^MCH^ neurons at the start of the dark phase (waking period) produces a dramatic increase in sleep duration, NREM sleep by 60% and REM sleep by 95% across the first 6 h [144]. Whereas optogenetic activation of MCH neurons during NREM sleep did not change the duration of NREM sleep but instead increased the probability of a transition from NREM sleep to REM sleep, and MCH neuron activation at the onset of REM sleep resulted in longer periods of REM sleep [145,146]. In order to investigate the downstream structures involved in this modulation of REM sleep, Jego, Glasgow, Herrera, Ekstrand, Reed, Boyce, Friedman, Burdakov and Adamantidis [145] also used optogenetics to target MCH fibers innervating the medial septum and tuberomammillary nucleus and found that activating these innervating fibers produced the same REM extension exhibited following optogenetic stimulation of the MCH neurons themselves. These results support the role of MCH in promoting REM sleep.

### 4.3. Dopaminergic Neurons

Optogenetic investigation of DA neurons in the VTA has shown that semi-chronic stimulation of VTA^DA^ neurons over 6 h is sufficient to maintain wakefulness and inhibit sleep-relevant behaviors such as nest building [40]. Conversely, when these neurons were inhibited animals exhibited nest building behaviors, suggesting that quieting these neurons triggers behaviors linked to sleep preparation [40]. Additionally, using fiber photometry to observe fluorescent calcium signals emitted by activation of Th*+* VTA neurons, Eban-Rothschild, Rothschild, Giardino, Jones and de Lecea [40] observed that these neurons are more active during waking and REM sleep than NREM sleep, showed a decrease in activity immediately prior to wake-to-NREM transitions, and showed an increase in activity prior to either NREM-to-REM transitions or NREM-to-wake transitions, as had been shown previously [39]. In line with the results of inhibiting these neurons it was also observed that these Th+ VTA neurons were quiet during nest building behaviors [40]. Similarly, investigation of DA neurons in the dorsal raphe nucleus with fiber photometry showed increased activity to both rewarding and aversive salient stimuli, and increased activation during waking and REM sleep compared to NREM [147]. Similarly to the VTA, optogenetic activation of dorsal raphe nucleus (DRN) DA neurons promotes wakefulness, whereas their inhibition promotes sleeping [147]. Interestingly, fiber photometry results showed that VTA^DA^ neurons appeared to have increased activity during REM sleep compared to waking states [40], whereas DRN^DA^ showed higher activation during waking states compared to REM sleep, especially during the early stages of waking, showing a gradual decrease in activity across the waking phase [147]. Considering previous evidence from measurements of Hcrt1 in CSF suggesting that wake-promoting Hcrt neurons activity peaks in the latter third of the day [56], it is possible that different wake-promoting neurons populations modulate wake-states throughout the day, with DRN^DA^ neurons promoting wakefulness in the earlier stages of the waking period, and Hcrt neurons taking over modulation of arousal as the wake-phase progresses.

### 4.4. Cholinergic Neurons

Wake- and REM-active cholinergic neurons are found in the basal forebrain [25,26,30] and the mesopontine tegmentum [31,32,33,34,35]. Due to the heterogeneity of these areas results from lesion studies and electrophysiological stimulation studies have produced mixed results (as discussed in Section 3), however the use of optogenetics has provided more precise targeting of these neurons. Optogenetic activation of cholinergic basal forebrain neurons increases NREM-wake transitions, resulting in increased waking-duration and decreased NREM-duration [30], suggesting that these are a population of wake-promoting neurons. Interestingly, optogenetic activation of cholinergic neurons in the pedunculopontine tegmentum or the laterodorsal tegmentum increase the likelihood of REM sleep, and this increase in REM sleep is due to a greater number of REM sleep episodes, rather than an increase in the duration of the REM sleep episodes [148]. This suggests that cholinergic neurons in the pedunculopontine tegmentum and laterodorsal tegmentum are playing a role in the initiation of REM sleep episodes, rather than the maintenance of REM sleep episodes. These results suggest a wake-promoting role for cholinergic neurons in the basal forebrain, and a REM-initiation-promoting role for cholinergic neurons in the mesopontine tegmentum.

### 4.5. GABAergic Neurons

As previously stated, GABAergic neurons are located throughout the central nervous system and are co-localized with many different sleep-relevant neuropeptides. Optogenetic activation of GABAergic neurons found in the ventral medulla has been shown to rapidly and reliably induce REM sleep, or extend REM sleep episodes when stimulated during an already initiated REM sleep episode, whereas pharmacogenetic inhibition of these neurons results in dose-dependently reduced REM sleep [62]. Genetically targeted optrode recordings also showed that these ventral medulla GABAergic neurons are most active during REM sleep, and during wakefulness they responded preferentially to feeding and grooming behaviors [62]. Interestingly, optogenetic activation of GABAergic neurons in the bed nucleus of the stria terminalis (BdNST) during NREM sleep actually triggers transitions into wakefulness, however stimulating these neurons during REM sleep produces no transition from REM sleep [149]. Additionally, investigation of a group of GABAergic neurons in the LH that are known to project to the ventrolateral preoptic area, and do not appear to co-express either Hcrt or MCH showed that chemogenetic activation of these neurons produces increased waking-duration, whereas their inhibition causes increased sleep-duration [150]. Conversely, optogenetic activation of a population of GABAergic neurons in the preoptic area that project to the tuberomammillary nucleus resulted in increased NREM and REM sleep, and decreased waking, whereas their inhibition increased waking and decreased both NREM and REM sleep duration [151]. Taken together this suggests that LH and BdNST GABAergic neurons promote wakefulness, whereas preoptic area GABAergic neurons promote both NREM and REM sleep, and ventral medulla GABAergic neurons promote REM sleep.

A recent optogenetic investigation of the basal forebrain studied the role of two GABAergic neuron populations in sleep/wake states. It was observed that parvalbumin-positive GABAergic neurons are primarily wake- and REM-active, and optogenetic activation of these neurons results in increased NREM-wake transitions, and an overall increase in the duration of waking states and decrease in the duration of NREM sleep [30]. Conversely, somatostatin-positive GABAergic neurons showed more activity during NREM sleep states, and activation of these neurons promotes NREM sleep [30]. Further investigation revealed that the parvalbumin-positive GABAergic neurons received inhibitory connections from the somatostatin-positive GABAergic neurons [30]. These results suggest a possible circuit for controlling sleep-wake transitions in the basal forebrain via different neuron populations.

### 4.6. Astrocytes

While DA, Hcrt, MCH, and GABAergic neurons are well accepted as being part of the sleep-wake state machine, recent studies have also taken advantage of optogenetic tools to investigate possible roles in sleep regulation in other non-neuron cell types in the brain. Pelluru, et al. [152] used the promoter for the astrocyte-specific cytoskeletal protein (GFAP) to optogenetically target astrocytes to investigate the possibility of astrocyte regulation of sleep-wake states. Although a range of stimulation frequencies were used (0, 5, 10, 30 Hz), only 10 Hz stimulation of astrocytes produced a significant decrease in waking and a significant increase in both in REM and NREM sleep duration [152]. This finding suggests a possible role for astrocytes in the maintenance of sleep-states; however further investigation is required to understand the precise role of these cells, and the importance of a 10 Hz stimulation frequency.

## 5. Optogenetic Tools for Circuit Investigation

While optogenetic tools have provided insight into each of these neuron populations individually, the most beneficial quality of these tools is the possibilities they present for circuit investigation. By combining optogenetic methods with other pharmacological and genetic approaches such as DREADDs, chemogenetic inhibition/excitation, or genetic mouse models researchers can observe how manipulating one part of the sleep–wake state circuitry affects the relevant upstream/downstream targets. The majority of studies using optogenetic methods to investigate these neuropeptides have focused on the wake promoting neurons, with a particular focus on interactions with Hcrt neurons.

### 5.1. Hypocretin Interactions with Histamine

Hcrt and histamine are both wake-active neuron populations and the possibility of some kind of sleep-wake state mediating circuit was suggested by in vitro evidence that Hcrt neurons excite histamine neurons [153], and reports of low histamine levels in the CSF of narcolepsy patients, particularly those who are not taking medications to regulate their narcolepsy [154,155]. Low levels of histamine were also observed in the brains of narcoleptic dogs [156]. Additionally, administration (i.c.v.) of Hcrt 1 promotes wakefulness, and this effect is attenuated by interruption of histamine H_1_ receptor function either by application of an H_1_ receptor antagonist [157,158] or altered in via gene manipulation in knockout mouse models [159], and patch clamp electrophysiological recordings have also shown that infusion of Hcrt increases the activity of histaminergic neurons [158]. Conversely, while the wake promoting effect of Hcrt 1 was reduced in histamine H_1_ receptor knockout mice [159], optogenetic stimulation of Hcrt neurons in histidine decarboxylase (HDC) knockout mice was sufficient to increase the probability of a sleep-wake transition [141]. These findings suggest that there is some interaction between the hypocretin system and the histamine system and their modulation of arousal.

To observe Hcrt activation of histamine neurons more closely Schone, et al. [160] combined optogenetic stimulation with in vitro slice electrophysiology to observe the response profiles of histamine neurons to optogenetic stimulation of Hcrt neurons. Hcrt neuron stimulation resulted in fast postsynaptic currents in histamine neurons and a robust connection of Hcrt neurons to histamine neurons was observed (~60% of histamine neurons received connections from ~40% of Hcrt neurons) [160]. Subsequent investigation of the role of the Hcrt receptor 2 (HcrtR2) in this connectivity revealed that Hcrt activation of histamine neurons could produce two distinct phases of histamine neuron firing: an initial fast transient firing peak which is unaffected by HcrtR2 antagonism and is followed by a slow firing phase that increases linearly during constant Hcrt activation and is abolished when HcrtR2 is blocked [161]. The same combination of methods was used to observe targets of tuberomammillary nucleus (TMN) histamine neurons following optogenetic stimulation of histamine neurons. Results showed that stimulating histamine neurons results in disinhibition of the wake-active ventrolateral TMN by decreasing inhibitory GABAergic inputs to the vlTMN, whereas histamine activation resulted in the inhibition of activity in the sleep-active ventrolateral preoptic nucleus (VLPO) [162]. These observations of two phases of histamine activation differentially affected by the blocking of HcrtR2 could explain inconsistencies in the role of histamine in Hcrt modulation of arousal seen in previous studies.

### 5.2. Hypocretin Interactions with the Locus Coeruleus

The densest afferent projections from Hcrt neurons project to the wake-promoting locus coeruleus [163] and in vivo studies have shown that Hcrt injections into the LC promote wakefulness and reduce REM sleep [164,165]. Additionally, while mice lacking the Hcrt receptor show narcolepsy-like symptoms, targeted restoration of Hcrt receptors in the LC can reduce symptoms of chronic sleepiness and fragmented wakefulness [166]. Similarly to Hcrt neurons, optogenetic low frequency stimulation of LC neurons during sleep induces immediate sleep-wake transitions, and in awake mice it triggers increases in activity and total waking-duration [167]. Additionally, c-Fos stainings have shown that optogenetic activation of Hcrt neurons in the LH results in increased neural activity in the LC [141]. To investigate how these neurons interact with the Hcrt system Carter, et al. [168] carried out a dual optogenetic approach in which they could simultaneously stimulate Hcrt neurons in the LH and either inhibit or stimulate LC neurons. Using this method they found that bilateral inhibition of LC neurons blocked the Hcrt-mediated sleep-wake transitions normally induced by optogenetic stimulation. Conversely, concomitant optogenetic stimulation of Hcrt neurons and LC neurons enhances Hcrt-mediated sleep-wake transitions. While the Hcrt neurons and LC neurons have similar wake-promoting effects, the millisecond precision allowed using optogenetics has allowed researchers to investigate the temporal distinctions between these neuron populations. While optogenetic stimulation of LC neurons triggers rapid, reliable sleep-wake transitions within ~5 s, Hcrt neurons act over a longer 10–30 s to induce arousal [169]. It is possible that, while Hcrt modulates sleep-wake transitions according to homeostatic need, the LC which is known to be active during exposure to stressful stimuli [41] might play a role in enhancing Hcrt modulation of arousal when the animal is in a state of hyper-vigilance.

### 5.3. Hypocretin Interactions with Leptin

While the majority of arousal and sleep–wake state research focuses on transitions between waking, NREM, and REM sleep due to their clear delineations, there is also the possibility of subcategorizing wake-states. Although there are not currently widely accepted criteria to define categories of wakefulness, states of quiet-waking and active-waking are discussed frequently as well as the state following presentation of salient stimuli to induce either exploratory behaviors (rewards) or stress responses (aversive/fearful) stimuli. Hypocretin has been linked to states of hyper-arousal such as stress in a study by Bonnavion, et al. [170], which showed that extended phasic high-frequency optogenetic stimulation of Hcrt neurons produced multiple physiological and behavioral markers of stress including elevated plasma corticosterone concentrations, increased heart rate, increased blood pressure, and reduced exploratory behavior in the open field task. Interestingly, in food deprived animals optogenetic stimulation of Hcrt neurons resulted in a 3-fold increase in corticosterone concentrations above that of mice fed ad libitum [170], suggesting that food deprivation heightened the stress-response induced by Hcrt activation.

Hcrt is inhibited following food intake [114] and by biomarkers that are released following food intake such as leptin, glucose, and neuropeptide Y [115,117,118,119,120]. Of these compounds it has been shown that leptin, which is released in response to dietary fat intake, is behaviorally anxiolytic [171,172]. LepRb neurons are also known to be present in the lateral hypothalamic area, and directly innervate Hcrt neurons in the LH [173]. By combining optogenetic activation of Hcrt with leptin infusion into the lateral hypothalamic area, Bonnavion, Jackson, Carter and de Lecea [170] showed that Hcrt-mediated increase in corticosterone that is enhanced by food restriction, is attenuated by leptin administration. Then, to observe the direct effect of leptin receptor activation they optogenetically activated LepRb neurons during a behavioral restraint stress paradigm, which is known to trigger increased corticosterone release. Results showed that activation of LepRb neurons resulted in a suppression of Hcrt activity and resulted in decreased corticosterone concentrations [170]. Taken together, these results show that nutritional status modulates physiological and behavioral markers of stress via the Hcrt system, and Hcrt-induced stress can be attenuated by leptin, which is endogenously released following dietary fat intake.

### 5.4. Hypocretin Interactions with Melanin-Concentrating Hormone (Feat. GABA)

Optogenetic methods have also been used to investigate the apparent opposing functions of wake-promoting Hcrt neurons [140] and REM-promoting MCH neurons [145,146] in the lateral hypothalamus. Gene-deletion studies have also provided evidence for contrasting functions of these two neuron populations: deletion of Hcrt neurons results in increased sleepiness (as seen in narcolepsy) and weight gain [107,108], whereas deletion of MCH neurons results in increased hyperactivity and reduced bodyweight [174,175,176]. Recent research investigating the interactions between these neurons using combinations of pharmacological manipulation, optogenetic manipulation, and network-level calcium imaging in in vitro slice recordings have shown that bath-application of Hcrt in the LH results in the activation of approximately 30% of MCH neurons [177]. Further investigation showed that optogenetic activation of Hcrt neurons resulted in a rapid reduction of firing in approximately 80% of the MCH neurons recorded [177], however this effect was attenuated following application of a GABA_A_ receptor blocker (10 μm gabazine), suggesting a possible role for GABA in mediating interactions between MCH and Hcrt neurons [177]. Indeed, optogenetic activation of Hcrt neurons produces increased GABAergic tone in the LH (also seen following bath application of Hcrt [161]) and this effect is reduced following application of Hcrt receptor antagonists [177]. Taken together, these results show that Hcrt neural activity can mediate MCH neuron activity either by excitation or inhibition, and that these interactions may be mediated via GABA_A_ receptors.

## 6. Further Developments and Future Directions

Optogenetic stimulation dramatically improved the ability of researchers to stimulate specific populations of neurons without unintentionally stimulating neighboring neuron populations. This specificity can also be achieved for the recording of neural activity of targeted neurons populations without inadvertently recording activity from other adjacent populations by using fiber-photometry [178,179]. The implementation of this method will be crucial for understanding endogenous function of neuron populations—without first observing the naturally occurring activity patterns of these neuron populations, the optogenetic stimulation of them will only allow researchers to understand what artificial activation of the neurons produces. Greater insight into the naturally occurring neural activity patterns of different types of neurons will be important for driving further studies manipulating these activity patterns using optogenetic stimulation.

The main improvement that optogenetic tools provide over traditional electrophysiological recording and stimulating methods is the ability to target specific neurons based on their gene-expression. However, this does not help if the population of neurons being studied is not yet genetically defined. Therefore, in order for researchers to receive the maximum benefit from these tools it is important to carry out a systematic characterization of the target population. Recent developments in next-generation sequencing methods now allow researchers to investigate gene-expression profiles of single neurons [180]. These methods will be extremely useful for defining subpopulations of neurons within highly heterogeneous structures, such as the hypothalamus, to allow greater specificity when genetically targeting neurons. In particular, the use of drop-seq to sequence the RNA of a single cell separated into nanoliter-sized droplets has now been used to successfully define 50 genetically distinct neuron types in the heterogeneous hypothalamic arcuate–median eminence complex [181]. These different methods are impressive when used alone, but the real benefits for circuit investigation are apparent when they are used in combination. A recent study by [151] investigated GABAergic neurons in the preoptic area, beginning with viral tracing methods, combined EEG and EMG sleep state monitoring, optogenetic manipulation, and optrode recordings they found a population of sleep-active, sleep-promoting GABAergic neurons in the preoptic area that project to the tuberomammillary nucleus. Further investigations identified multiple candidate markers found within the neurons by using cingle cell RNA sequencing and translating ribosome affinity purification. With these candidate genes [151] were then able to use further optogenetic and pharmacogenetic stimulation experiments to show a role for each of these gene candidates in promoting sleep. By following this example of using a combination of these techniques, researchers will be able to define their target neuron populations, observe the endogenous neuronal activity with fiber-photometry, and then optogenetically manipulate it.

## 7. Conclusions

While the optogenetic investigations discussed here have all taken great steps toward understanding the mechanisms underlying how the brain modulates between REM, NREM, quiet waking, active waking, and hyper-arousal states, there is still much to be learned. Each of the neural populations discussed here hold their own interest for the function of sleep and wake states, however, no behavior as complex as the modulation of sleep-wake transitions occurs in a single-structure vacuum, and it will only be by investigating circuits that researchers can finally comprehend the mechanisms via which animals can modulate sleep behaviors. Optogenetic technologies have vastly improved the ability of researchers to investigate the complex circuitry underlying sleep behaviors and these technologies will be fundamental in increasing our current knowledge of the sleep functions carried out in the brain. While much attention has been paid to optogenetic stimulation in particular, without first observing the endogenously occurring neuronal activation it would be impossible to successfully mimic neural activity using optogenetic stimulation. Therefore preliminary studies using optogenetic techniques such as fiber photometry should be taken advantage of to first observe the naturally occurring neural patterns researchers wish to recreate in the neurons expressing their neuropeptide of interest.

## Abbreviations

BdNST	Bed nucleus of the stria terminalis
CSF	Cerebrospinal fluid
DA	Dopaminergic
DREADD	Designer receptors exclusively activated by designer drugs
DRN	Dorsal raphe nucleus
EEG	Electroencephalogram
EMG	Electromyogram
GABA	γ Amino butyric acid
GFAP	Glial fibrillary acidic protein
Hcrt	Hypocretin
HcrtR2	Hypocretin receptor 2
Hz	Hertz
i.c.v.	Intracerebroventricular
LC	Locus coeruleus
LH	Lateral hypothalamus
MCH	Melanin-concentrating hormone
mRNA	Messenger ribonucleic acid
NE	Norepinephrine
NPS	Neuropeptide S
NREM	Non-rapid eye movement
REM	Rapid eye movement
TMN	Tuberomammillary nucleus
Vglut2	Vesicular glutamate transporter 2
VLPO	Ventrolateral pre-optic nucleus
vlTMN	Ventrolateral tuberomammillary nucleus
vPAG	Ventral periaqueductal gray
VTA	Ventral tegmental area
